# Intensification of induction chemotherapy before consolidation chemoradiotherapy improves progression-free survival and time without treatment in patients with locally advanced pancreatic cancers

**DOI:** 10.18632/oncotarget.25877

**Published:** 2018-08-10

**Authors:** Nicolas Williet, Thomas Di Bernardo, Chloé Vernet, Léa Saban Roche, Thierry Muron, Xavier Roblin, Nicolas Magne, Jean-Marc Phelip

**Affiliations:** ^1^ Hepatogastroenterology Department, University Hospital of Saint-Etienne, Saint-Etienne, France; ^2^ EA 7425 HESPER, Health Services and Performance Research, Claude Bernard Lyon 1 University, Lyon, France; ^3^ Medical Oncology Department, Institut Cancérologie Lucien Neuwirth, Saint-Priest-en-Jarez, France; ^4^ Radiotherapy Department, Institut Cancérologie Lucien Neuwirth, Saint-Priest-en-Jarez, France

**Keywords:** induction chemotherapy, locally advanced, pancreatic carcinoma, radiotherapy, survival

## Abstract

**Aims:**

To assess the interest of induction chemotherapy (ICT) intensification before chemoradiotherapy (CRT) in patients with locally advanced pancreatic cancer.

**Methods:**

Charts of patients treated between February 2010 and November 2016 with consolidation capecitabin based-CRT were retrospectively reviewed in this bicentric study. Patients who underwent Gemcitabine as ICT (Group G) were compared to patients treated with intensive ICT (group I). Primary objectives were progression-free survival (PFS), defined as the time from the first day of ICT to progression or last follow-up, and Time without treatment (TWT), as the time from the last day of CRT to progression.

**Results:**

Patients’ characteristics were balanced between group I (Folforinox: *n* = 24; GemOx: *n* = 6) and group G (*n* = 16) including mean age (63.7 vs 68.1 years), and performance status (PS 0-1 :90% vs 93.7%). Median PFS (17.8 months vs 12 months; *p* = 0.02) and TWT (7.4 months vs 2.5 months *p* = 0.01) were statistically better in group I vs group G. These results remained statistically and clinically significant by comparing Folfirinox subgroup to Gemcitabine. A trend to a better median overall survival was observed in group I (20.4 months) vs group G (18.3 months; *p* = 0.07). After adjusting for ICT duration, PS, and CA19.9 level, ICT intensification remains independently prognostic. Toxicity profile was in accordance with Literature.

**Conclusion:**

This study shows ICT intensification before CRT is an interesting approach in patients with locally advanced pancreatic cancer. Further studies are needed to confirm these results, and to assess the specific role of CRT in this setting.

## INTRODUCTION

Pancreatic ductal adenocarcinoma (PDAC) will be the second-leading cause of cancer-related deaths in Europe and in the United-States in 2030 [1, 2]. The majority are metastatic at the time of diagnosis and about 30% are locally advanced, mainly due to arterial involvement. However, this setting remains heterogenous as it regroups potentially resectable tumors and definitely unresectable tumors. These two subgroups are yet to be defined using set guidelines. The treatment's objective, however, may be different in clinical practice. In the first subgroup, 26% of patients treated with FOLFIRINOX (leucovorin and fluorouracil plus irinotecan and oxaliplatin) experience a reduction in tumor size allowing tumor resection [3] and experience similar overall survival (OS) to those with tumor that are immediately resectable [4]. With these results in mind, tumor response rate could be the primary endpoint for these patients, and achieve up to 30% with FOLFIRINOX [3] vs 10% with Gemcitabine alone [5, 6]. In contrast, if the tumor remains unresectable after induction chemotherapy (ICT), primary objectives of the treatment should be progression-free survival (PFS) and overall survival (OS), with an acceptable toxicity profile.

Level of evidence is low regarding management of these patients. Hence, chemotherapy based Gemcitabine alone remains the standard of care for patients with locally advanced pancreatic cancer in Europe and United-states [7, 8]. After achieving disease-control with Gemcitabine based chemotherapy, capecitabine-based chemoradiotherapy (CRT) is then an acceptable therapeutic option in the goal of potentially improving the local-control rate and the time without treatment (TWT) [9], even if no benefit in terms of OS has been provided in the phase III trial LAP07 [9].

However, in the metastatic setting, two phase III randomized clinical trials showed the superiority of an intensive chemotherapy such as FOLFIRINOX or Gemcitabine + Nab-paclitaxel to Gemcitabine alone in terms of survivals and quality of life [10, 11]. But, these results have not been evaluated yet for locally advanced PDAC. Moreover, exposing these patients, who have an 18 months median OS, to Folfirinox-related toxicity, such as distal dysesthesia or hypoesthesia, from the first day of treatment to death, may raise some questions. Hence, a growing number of prospective studies are currently carried out for assessing the benefit of chemotherapy intensification in patients with locally advanced PDAC [12–14]. In NEOPAN, a phase III study, patients are randomized between Gemcitabine vs FOLFIRINOX. Pending these results, we carried out the present study to assess retrospectively the benefit of induction chemotherapy intensification compared to Gemcitabine alone in terms of survivals and TWT, in patients with locally advanced PDAC definitively unresectable and who were fit for consolidation CRT.

## RESULTS

During the period study, 144 patients with borderline or locally advanced PDAC were treated in our two centers (Flow-chart in Supplementary Figure 1): 47 with Gemcitabine alone, 56 with Folfirinox, and 17 with Gemox. Twenty-four additional patients underwent either another chemotherapy schedule or best supportive care. During the induction chemotherapy period, a total of 74 patients were excluded for disease progression (*n* = 53), secondary surgery (*n* = 15), or for PS ≥ 2 (*n* = 5). One death due to pulmonary embolism was observed in the Folfirinox subgroup. The 46 remaining patients were included in the population study.

### Characteristics of the population study at baseline

All patients and tumor characteristics at baseline were reported in Table 1. Of the 46 patients who were included, 16 (34.8%) were treated with Gemcitabine alone and 30 (65.2%) with Intensive ICT (GEMOX: *n* = 6, FOLFIRINOX: *n* = 24). Patients were comparable for age (mean: 68.1 yrs ± 8.5 and 63.7 yrs ± 6.4; *p* = 0.08), proportions of women (50% vs 46.7%; *p* = 1.0), cardiovascular history including ischemic myocardiopathy (12.5% vs 3.3%; *p* = 0.53), performance status (PS 0: 62.5% vs 80%; *p* = 0.08), and blood levels of bilirubin (median: 11 μmol/l [7.8–18.6]), albumin (40.1g/L [37.6–43]) and CA19.9 (222.5 UI/L [45–452.8] vs 96.2UI/L [36.3–384.8]; *p* = 0.79).

**Table 1:** Characteristics of patients at baseline

	Total population (*n* = 46)	Gemcitabine group (*n* = 16)	Intensive ICT group (*n* = 30)	*P* value
Age, mean (± SD)	65.3 (± 7.4)	68.1 (± 8.5)	63.7 (± 6.4)	0.08
Female	22 (47.8%)	8 (50%)	14 (46.7%)	1
Medical history				
arterial hypertension	20 (43.5%)	9 (56.2%)	11 (36.7%)	0.38
Diabetes mellitus	15 (32.6%)	7 (43.8%)	8 (26.7%)	0.41
Ischemic myocardiopathy	3 (6.5%)	2 (12.5%)	1 (3.3%)	0.53
Peripheral artery disease	2 (4.3%)	1 (6.2%)	1 (3.3%)	1
Cerebrovascular accident	2 (4.3%)	1 (6.2%)	1 (3.3%)	1
Performance status at baseline				
0	34 (73.9%)	10 (62.5%)	24 (80%)	0.08
1	8 (17.4%)	5 (31.2%)	3 (10%)	
Bilirubine at baseline, median [IQR25-75] (NA = 19)	11 [7.8–18.6]	11 [10–32]	11.8 [7.3–18.3]	0.68
Albumine, median [IQR25-75] (NA = 28)	40.1 [37.6–43]	40.2 [35.1–43]	40 [37.8–42.5]	1
Tumor Localization				
Head/isthmus/Winslow	39 (84.8%)	15 (93.8%)	24 (80%)	0.39
Body/tail	7 (15.2%)	1 (6.2%)	6 (20%)	
Tumor differenciation				0.08
Well to moderate	30 (65.2%)	7 (100%)	23 (76.7%)	
Low	7 (15.2%)	0 (0%)	7 (23.3%)	
CA19.9 at baseline, median [IQR25-75] (NA = 14)	116.6 [36–388.2]	222.5 [45–452.8]	96.2 [36.3–384.8]	0.79
CEA at baseline, median [IQR25-75] (NA = 22)	2.8 [1.7–5]	2.2 [1.8–4]	3.2 [1.6–5.4]	0.87

Abbreviations: ICT: induction chemotherapy; IQR: interquartiles range; NA:Not available; SD: standard deviation

Tumor was located in the head or isthmus of the pancreas in the vast majority (84.8%). In contrast, there was a trend to less low-differenciated tumor in Group G (0.0%) compared to Group I (23.3%; *p* = 0.08). Similarly, median tumor size tended to be smaller in group G (30 mm [23–36]) vs group I (35 mm [30–42]; *p* = 0.08)

### Characteristics at the end of induction treatment

Corresponding characteristics at the end of induction treatment were reported in Table 2. The following data were similar between the two groups: median ICT duration (3 months [2.7–4.5] vs 4.2 months [2.1–5.7]; *p* = 0.55), total duration of induction treatment including CRT (6.2 months [5.3–8.1] vs 6 months [5.6–8.7]; *p* = 0.63), median level of bilirubin (6 μmol/L [5–9] and 6 μmol/L [4.5–8.8]; *p* = 0.78), albumin (34.5 g/L [33.1–35.8] vs 36.3 g/L [33.8–40]; *p* = 0.67), CA19.9 levels (108.7UI/L [36.8–494.2] vs 57UI/L [24.1–224.6]; *p* = 0.85), CA19.9 variation from baseline (−165.5 [−278.8–−65.9] vs −3.4 [−257–+20.4]; *p* = 0.15), and tumor response rate (18.8% vs 20.0%; *p* = 1.0).

**Table 2:** Characteristics of patients at the end of induction chemotherapy

	Total population (*n* = 46)	Gemcitabine group (*n* = 16)	Intensive ICT group (*n* = 30)	*P* value
Duration of ICT, months, median [IQR25-75]	3.7 [2.5–5.5]	3 [2.7–4.5]	4.2 [2.1–5.7]	0.55
Performance status at the end of ICT				0.08
0	23 (50%)	6 (37.5%)	17 (56.7%)	
1	15 (32.6%)	7 (43.8%)	8 (26.7%)	
2	1 (2.2%)	1 (6.2%)	0 (0%)	
Bilirubin (NA = 22), median [IQR25-75]	6 [4.8–9.2]	6 [5–9]	6 [4.5–8.8]	0.78
Albumin (NA = 37), median [IQR25-75]	36.3 [33–37.7]	34.5 [33.1–35.8]	36.3 [33.8–40]	0.67
CA19.9 at the end of ICT (NA = 25), median [IQR25-75]	57 [25.9–249.7]	108.7 [36.8–494.2]	57 [24.1–224.6]	0.85
Delta CA19.9 (NA = 22), median [IQR25-75]	−46.9 [−269.8–7.7]	−165.5 [−278.8–−65.9]	−3.4 [−257–20.4]	0.15
RECIST at the end of ICT				1
Progression	3 (6.5%)	1 (6.2%)	2 (6.7%)	
Tumor response	9 (19.6%)	3 (18.8%)	6 (20%)	
Stable	34 (73.9%)	12 (75%)	22 (73.3%)	
Time for ICT + CRT, mean (± SD)	7.1 (± 2.5)	6.6 (± 1.9)	7.3 (± 2.8)	0.32
median [IQR25-75]	6 [5.5–8.6]	6.2 [5.3–8.1]	6 [5.6–8.7]	0.63
Number of chemotherapy Cycles, Median [IQR25-75]	−	4 [3.5–6.0]	7 [6–11.7]	−
Dosimetry, mean (± SD)	48.2 (± 5.6)	48.5 (± 7.4)	48.1 (± 4.4)	0.85
median [IQR25-75]	50.4 [45–50.4]	50.4 [50.4–50.4]	50 [45–50.4]	0.1

Abbreviations: CRT: chemoradiotherapy; ICT: induction chemotherapy; IQR: interquartiles range; NA:Not available; SD: standard deviation.

However, there was a trend to more PS score 0 in Group I (56.7%) compared to the Group G (37.5%; armitage test *p* = 0.08). In contrast, median total radiation dosimetry trend to be lower in the corresponding groups (50Gy [45–50.4] vs 50.4Gy [50.4–50.4], respectively; *p* = 0.1). Median number of chemotherapy cycles was 7 cycles [6–11.7] and 4 cycles [3.5–6]), respectively.

### Tolerance of the treatment

**Figure 1:**
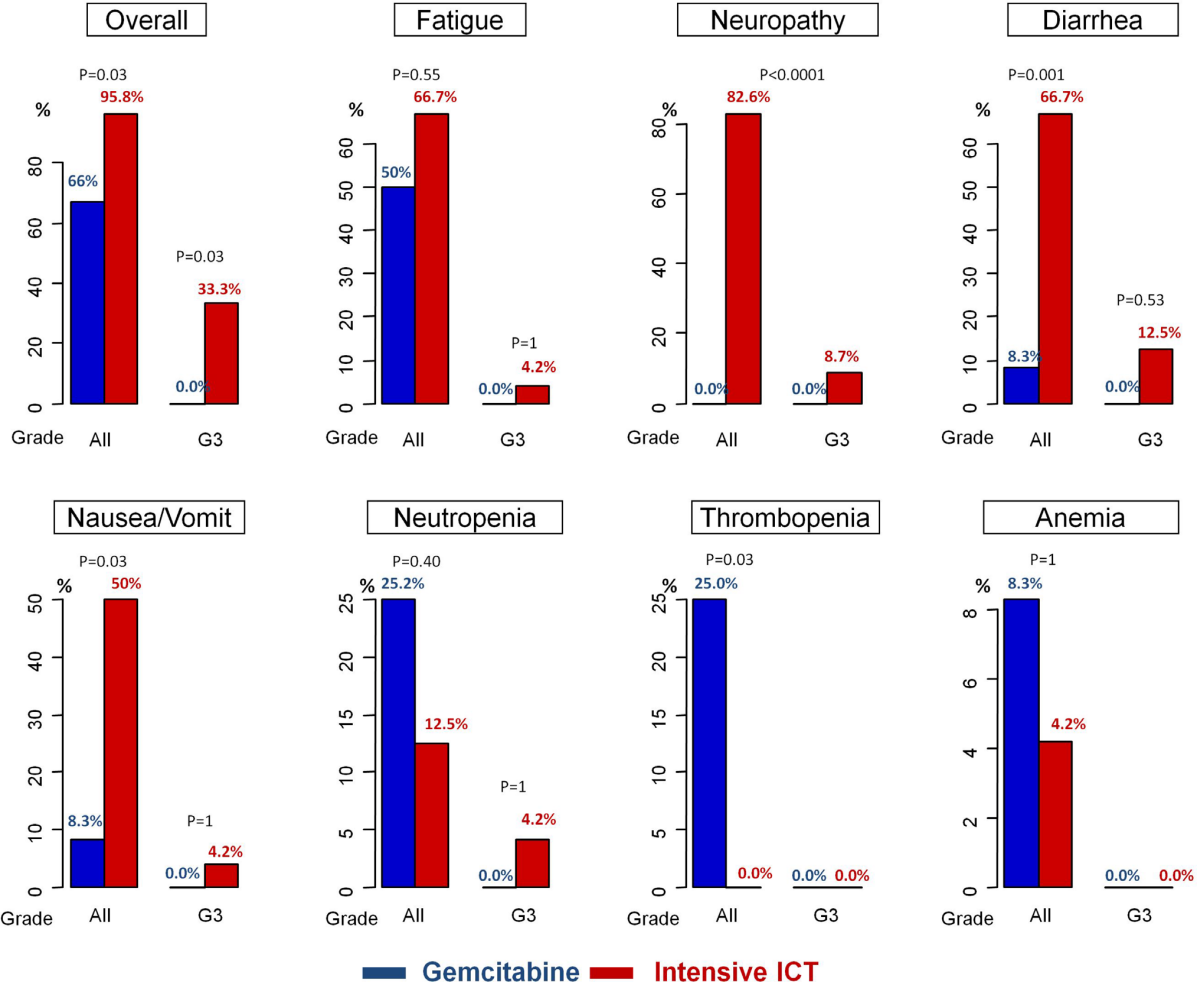
Chemotherapy-related toxicities

All side-effects related to ICT were reported in Figure 1. There was no grade 4 toxicity in both groups. Only Group I experienced grade 3 toxicities for diarrhea (12.5%), alopecia (10.0%), oxaliplatin-induced neuropathy (8.7%), fatigue (4.2%) and neutropenia (4.2%). Only grade 3 for overall toxicity was significantly higher in Group I (33.3% vs 0.0%; *p* = 0.03). Any grade toxicity was higher in group I vs group G regarding overall toxicity (95.8% vs 68%; *p* = 0.03), diarrhea (66.7% vs 8.3%; *p* = 0.001), and nausea/vomit (50% vs 8.3%; *p* = 0.03). In contrast, no thrombopenia was reported for group I (0.0%) vs 25% of the patients treated with Gemcitabine (*p* = 0.03). There is no difference statistically significant between the two groups for any grade fatigue (66.7% vs 50%), neutropenia (12.5% vs 25.2%), or anemia (4.2% vs 8.3%).

### Survivals

**Figure 2:**
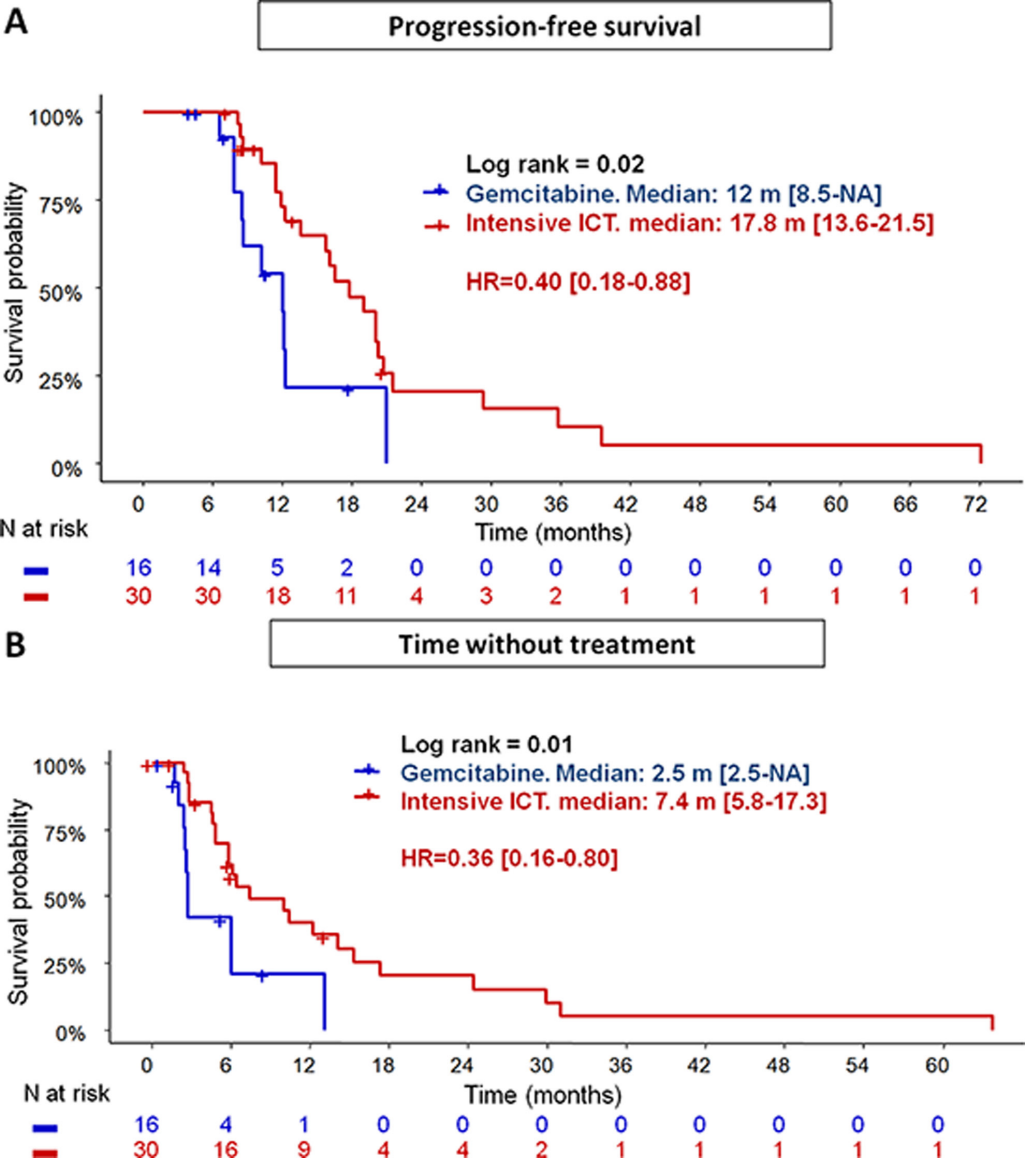
Progression-free survival (**A**) and time without treatment (**B**) in patients treated with intensive chemotherapy induction (Gemox or Folfirinox) vs Gemcitabine.

Thirty three progressions (Group G: 10; Group I: 23) were observed during a median 15.5 months follow-up. PFS (Figure 2A) was significantly superior in the Group I (median: 17.8 months; 95% CI: 13.6–21.5) compared to Group G (12 months: 95% CI: 8.5-NA), corresponding to a reduction of the progression-risk over time by 60% (HR = 0.40; 95% CI: 0.18–0.88; *p* = 0.02). These results remained statistically and clinically significant regarding the subgroup of patients treated with Folfirinox (17.8 months; 95% CI: 13.6–20.7) compared to those treated with Gemcitabine alone (12.0 months; 95% CI:8.5-NC; *p* = 0.037) (Supplementary Figure 2A). Only ICT intensification (group G vs group I) was prognostic after univariate analyses. After adjusting on ICT duration, PS and CA19.9 level at baseline, PFS remains better in group I vs Group G: HR = 0.12 (95% CI: 0.03–0.55); *p* = 0.006.

**Figure 3:**
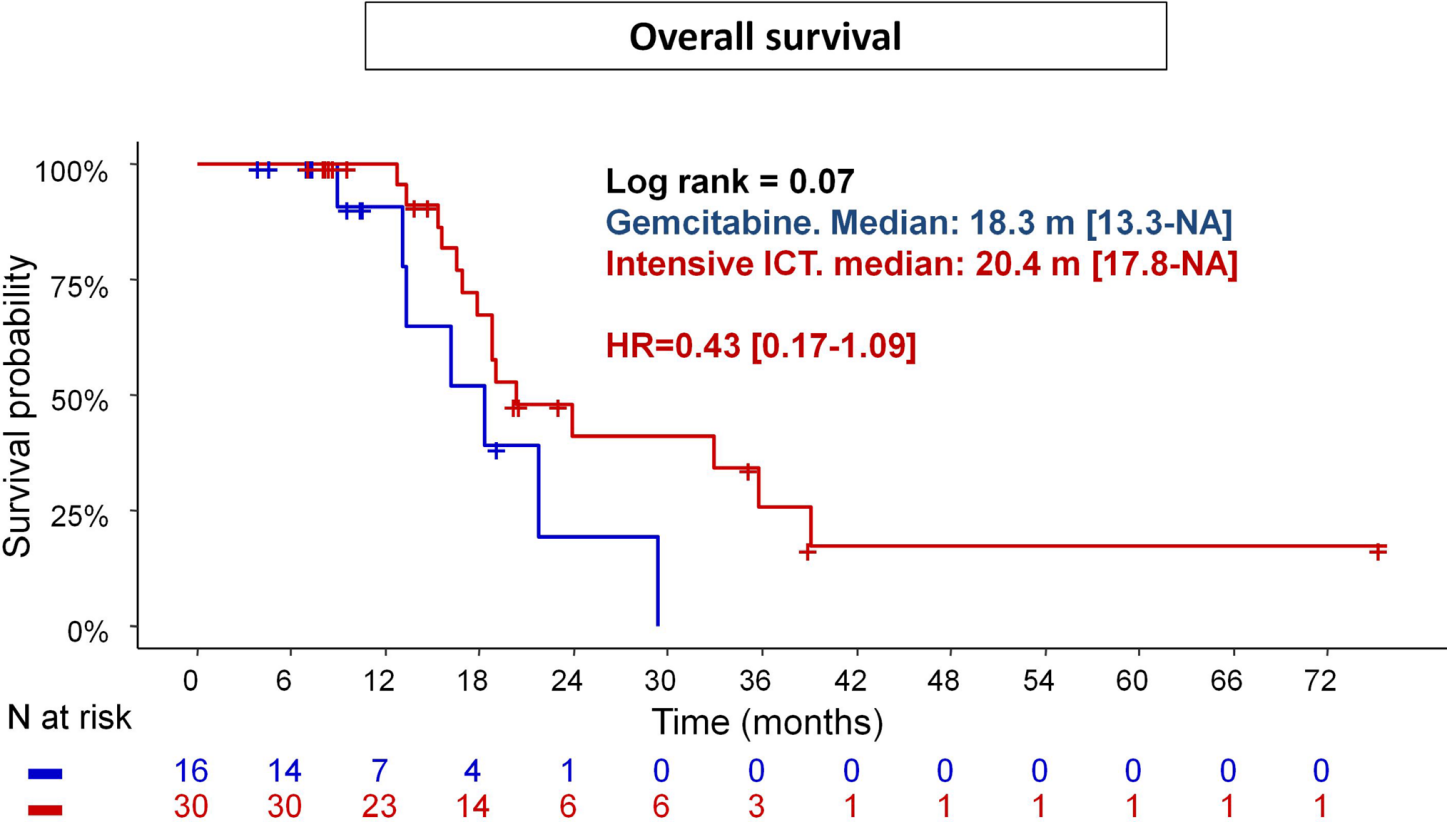
Overall survival in patients treated with intensive chemotherapy induction (Gemox or Folfirinox) vs Gemcitabine

Twenty two deaths (Group G: 7; Group I: 15) occurred during follow-up. Median OS (Figure 3) tended to be higher in the Group I (20.4 months; 95% CI: 17.8-NA) compared to Group G (18.3 months; 95% CI: 13.3-NA), corresponding to a reduction of death-risk over time by 57% (HR = 0.43; 95% CI: 0.17-1.09; *p* = 0.08). This trend was not confirmed in Folfirinox subgroup (median OS: 19.0 months; 95% CI: 16.9–NC; *p* = 0.21 (Supplementary Figure 3). There is no other factor impacting on OS.

### Time without treatment (Figure 2B)

From the last day of CRT, median TWT was higher in Group I (7.4 months; 95% CI: 5.8–17.3) compared to Group G (2.5 months; 95% CI: 2.5-NA; *p* = 0.01), corresponding to a reduction over time by 64% (HR = 0.36; 95% CI: 0.16–0.80). These results remained statistically and clinically significant by comparing subgroup of patients treated with Folfirinox to those with Gemcitabine alone (median TWT): 6.4 months (5.8–15.3) vs 2.7 months (2.5-NC); *p* = 0.028 (Supplementary Figure 2B). Univariate analyses showed ICT intensification (*p* = 0.01), bilirubin level at the end of CRT (*p* = 0.004), total dosimetry (*p* = 0.1) and number of fractions (*p* = 0.13) were prognostic for the TWT. These variables, adjusted on duration of induction period and PS at the last day of ICT, remain statistically prognostic (Table 3), with a negative impact on the TWT by cumulated radiation dose (HR = 1.73; 95% CI: 1.03–2.9; *p* = 0.04), and bilirubin level (HR = 1.23; 95% CI: 1.09–1.41; *p* = 0.001). In contrast, intensive ICT (HR = 0.05; 95% CI: 0.01–0.34; *p* = 0.003), and number of fractions (HR = 0.37; 95% CI: 0.15–0.95; *p* = 0.04) were positive prognostic factors. Of the 33 disease-progressions observed within follow-up, 21 involved at least the primitive tumor (63.6%). Local progression rates tended to be higher in the Group G (90.0%) vs Group I (59.1%; *p* = 0.11).

**Table 3:** Uni- and multivariate analyses by cox regression for time without treatment

Univariate analyses			Multivariate analyses	
Variables	*P* value	HR [95% CI]	*P* value	HR (95% CI)
**Clinic parameters at baseline**				
Age	0.48	1.02 [0.97–1.07]		
Sex	0.97	0.98 [0.47–2.06]		
arterial hypertension	0.44	1.32 [0.65–2.69]		
Diabetes mellitus	0.97	1.01 [0.48–2.16]		
Ischemic myocardiopathy	0.56	1.54 [0.36–6.59]		
Peripheral artery disease	0.95	1.07 [0.14–8.03]		
Cerebrovascular accident	0.99	0 [0–Inf]		
Performance status	0.7	0.78 [0.23–2.7]		
**Biologic parameters at baseline**				
Bilirubin level	0.60	1 [0.98–1.01]		
Albumine level	0.27	1.06 [0.96–1.18]		
CA19.9	0.34	1 [1–1]		
CEA	0.56	0.97 [0.87–1.08]		
**Tumor characteristics**				
Tumor differenciation	0.36	0.59 [0.19–1.83]		
Tumor localization	0.32	1.63 [0.62–4.29]		
**Induction therapy schedule**				
Intensive ICT	0.01	0.36 [0.16–0.8]	0.003	0.05 [0.01–0.34]
Time for ICT	0.99	1 [0.82–1.22]	0.68	1.07 [0.77–1.51]
Dosimetry	0.1	1.08 [0.99–1.17]	0.04	1.73 [1.03–2.9]
Number of fraction	0.13	1.13 [0.96–1.32]	0.04	0.37 [0.15–0.95]
Time for CRT	0.64	1.04 [0.89–1.21]		
Center	0.94	0.97 [0.48–1.98]		
**Induction therapy tolerance to the end of ICT period**				
Performance status	0.92	1.04 [0.49–2.2]	0.052	0.19 [0.04–1.01]
Grade 3-4 Overall toxicity	0.32	0.83 [0.58–1.19]		
Bilirubin level	0.004	1.11 [1.03–1.19]	0.001	1.23 [1.09–1.41
Albumine level	0.66	0.97 [0.82–1.13]		
**Tumor response at the end of ICT period**				
CA19.9	0.51	1 [1–1]		
Delta CA19.9 from baseline	0.34	1 [1–1]		
Tumor response (RECIST)	0.96	1.03 [0.27–3.91]		
Stability disease (RECIST)	0.18	0.43 [0.12–1.49]		

### Treatment at progression

Regarding treatment at progression, data missed for 6 patients in both groups. In group G, 3 patients could be treated with a second line therapy such as FOLFIRI (*n* = 1) or FOLFOX (*n* = 2). In group I, 6 patients resumed a lighter front line regimen (subgroup FOLFIRINOX: *n* = 3 FOLFOX; *n* = 2 FOLFIRI) (subgroup GEMOX: *n* = 1 Gemcitabine alone). Twelve other patients underwent a second-line therapy (subgroup FOLFIRINOX: *n* = 6 Gemcitabine alone; *n* = 3 Gemcitabine + Nab-paclitaxel) (Subgroup GEMOX: *n* = 2 Folfirinox, *n* = 1 Folfiri). Overall, patients could be treated with chemotherapy at progression in a statistically higher proportion in the Group I (60.0%) compared to the Group G (16.6%; *p* = 0.02).

## DISCUSSION

Management of patients with locally advanced PDAC remains debatted. Indeed, the level of evidence remains low in this setting. Induction chemotherapy by Gemcitabine is currently the standard of care. However, intensive chemotherapy schedules (FOLFIRINOX or Gemcitabine + Nab-Paclitaxel) are frequently used in clinical practice, because their superiority to Gemcitabine alone was well demonstrated in two phase III trials either in terms of PFS or OS [10, 15]. The second reason of intensification ICT in clinical practice is probably the secondary resection rates that achieve up to 26% with FOLFIRINOX [3] or 15% to 28.6% with the combination Gemcitabine + Nab-Paclitaxel [13, 14]. Hence, a growing number of prospective studies are evaluating these schedules in this setting. To date, the two largest prospective studies evaluating FOLFIRINOX in patients with locally advanced PDAC showed a median overall PFS ranging from 13 to 16 months and a median OS from 22 to 25 months [16, 17]. About 2/3 of these patients underwent consolidation CRT. Hence, results of our study is in accordance with literature. However, our population was more selected because we excluded all patients who underwent secondary surgery after tumor shrinkage with ICT, those who were not fit for consolidation CRT at the end of the induction period and all patients who experienced disease-progression within induction period. This method of selection allowed to make well-balanced the two groups regarding tumor characteristics, its potential evolution during the induction period and patients’ characteristics. Indeed, all patients who are fit for GEMCITABINE alone are not necessarily fit for FOLFIRINOX, nor for GEMOX. Due to the retrospective nature of the present study, non-intent-to-treat analysis was conducted to make the two groups as similar as possible. However, despite the non statistical significance, absolute values of PS-0 (62.5% in the group G vs 80% in group I) and CA 19-9 (median: 222.5 vs 96.2UI/L) remain important and are very powerfull negative prognostic factors for survival. But, there is a relevant number of missed-data regarding CA19.9 value. Moreover, ICT intensification remains independently prognostic after adjusting for ICT duration, PS, CA19.9 value at the end of ICT.

Hence, we showed for the first time the yield of ICT intensification to Gemcitabine alone in terms of PFS and more interestingly regarding TWT. Indeed, TWT (or time to progression from the end of CRT) appears to us a more relevant objective for these patients to improve their quality of life. Moreover, chemotherapy-related toxicities were manageable, including rare grade 3 oxaliplatin-induced neuropathy (8.7%), and more fatigue in Group I compared to Group G without reaching the statistical significance. Interestingly, these related-chemotherapy toxicities did not reduce the possibility of treatment resumption, and OS tended to be higher in Group I although there were more low-differentiated tumor in this group.

The main limit of our study is its small size, thereby reducing the statistical power to demonstrate that Intensive ICT induced more any grade fatigue and grade 3 diarrhea. In contrast, despite this limit, survival results of the present study are clinically and statistically in favor of ICT intensification which support current clinical practice. Due to this limit we also failed to demonstrate the improvement of local-progression rates observed in favor of the ICT intensification (59.1% vs 90.0%; *p* = 0.11). However, this point is crucial as we know how such local-progression can limit or delay treatment resumption in clinical practice. Then, this difference in terms of local-progression rates may also explained the statistically lower rate of treatment resumption observed in Group G vs group I (16.6% vs 60.0%; *p* = 0.02).

Another limit of the present study is its retrospective nature and the selection method that is not in intent-to-treat. Hence, exclusion of patients presenting a tumor shrinkage allowing surgery as well as patients with tumor progression during the induction period represent a major bias. Moreover, the period of inclusion is long and is overlapping with a strong change of clinical practice, as Folfirinox was validated in 2011. Some of patients (*n* = 6) from the group I were treated by GEMOX, basing on results from previous phase III randomized clinical trials that showed improvement of overall response rate (27–31%) with GEMOX vs Gemcitabine alone (4–15%). In our study, half of these patients were treated in 2011 that is before PRODIGE4/ACCORD11 publication [10]. The 3 remaining patients who were treated after 2011 were probably not fit for FOLFIRINOX. However, although GEMOX remains a therapeutic option according to the National Comprehensive Cancer Network^®^ (NCCN^®^) guidelines [7], its use has become rare in this setting in clinical practice because no benefit was demonstrated in metastatic setting regarding overall survival [6, 18].

In our study, no patient was treated with the Nab-paclitaxel + Gemcitabine combination. In France, Nab-Paclitaxel is not refunded. Only a few number of centers such ours can funding this nano-drug but only for the treatment of patients with PDAC in metastatic setting. Recent prospective studies, such as LAPACT [14], have provided promising survival results with this combination therapy in locally advanced PDAC: about 8 months for the median time to treatment failure. Some of these patients underwent CRT. The remaining patients continued the combination until progression or underwent laparotomy to assess whether surgical resection of the tumor was feasible (15%). Another phase II prospective study, NEOLAP, randomized patients to Gemcitabine + Nab-Paclitaxel or FOLFIRINOX in this setting: interim results were recently reported and secondary resectability in locally advanced PDAC appeared similar between the two groups [13]. Finally, only results from the phase III study NEOPAN [12] will provide robust data pros or cons the intensification of ICT in patients with locally advanced PDAC. Pending, our study provides encouraging results supporting this approach.

## MATERIALS AND METHODS

### Population study

All patients with locally advanced PDAC, treated consecutively between February 2010 and November 2016 in the University hospital of Saint-Etienne or in the Institut Cancérologie Lucien Neuwirth were retrospectively reviewed. Only patients who underwent induction chemotherapy for 2 to 6 months followed by Capecitabine-based chemoradiotherapy (830 mg/m2 orally, twice daily on days 1–21 of a 28 day cycle) were included. CRT was proposed only if disease-control was achieved and if patient's PS ranged from 0 to 1. Hence, all patients who experienced tumor progression within the induction period were excluded as well as those who achieved tumor shrinkage allowing secondary resection.

Two groups of patients were compared, according to the intensity level of ICT: the first one (Group G) involved patients who were treated with Gemcitabine alone (1000 mg/m^2^ weekly, 3 consecutive weeks on 4); the second one (Group I) involved patients who were treated with combination chemotherapy such as: Gemcitabine (1000 mg/m^2^) + oxaliplatin (100 mg/m^2^) [GEMOX] or 5 Fluorouracil (Bolus: 400 mg/m^2^; IV infusion: 2400 mg/m^2^ for 46-48H) + Leucovorin (400 mg/m^2^) + Oxaliplatin (85 mg/m2) + irinotecan (150–180 mg/m^2^) [FOLFIRINOX]. To make these groups as similar as possible, we also excluded patients who were not fit for consolidation CRT.

### Data collection

All clinical, biological, pathologic and radiological data were retrospectively collected from electronic patient's chart. Some informations, such events or some biological data before 2011, were extracted from paper charts. This study was approved by local ethical committee (IRBN632017/CHUSTE) and conducted in agreement with the «Loi informatique et libertés» (January 6, 1978, modified by the July 1, 1994 law and finalized by the August 6, 2004 law).

PFS was defined as the time from the first day of ICT to the time of disease-progression or last follow-up. Similarly, OS was defined as the time from the first day of ICT to death or last follow-up, and TWT as the time from the last day of CRT to the time of disease-progression or last follow-up.

### Statistical analyses

The primary objective of the present study was PFS and TWT. Secondary objectives were OS, chemotherapy-related toxicities, local-progression rates, and treatments at progression. Survivals were estimated using the Kaplan–Meier method and compared with Log-Rank tests. Prognostic factors were identified using Cox proportional hazards regression by adjusting on ICT duration, PS and CA19.9 level at the last day of CRT for TWT and on corresponding data at baseline for survivals.

Qualitative variables were reported as numbers and percentages, and compared by using chisq or fisher exact test as appropriate. Percentages of ordinal variables were compared using Cochrane–Armitatge test. Quantitative variables were reported as median value with their interquartile ranges from 25% to 75%, and compared by using Mann–Whitney wilcoxon test. Some of them were additionnaly reported as mean value with standard deviation (SD). All statistical analyses were performed using R, version 3.2.2 (R project, Auckland, New Zealand).

## CONCLUSIONS

The present study supports the benefit of ICT intensification in patients with locally advanced PDAC, both in terms of PFS and, more interestingly, for time without treatment. Chemotherapy-related toxicities were acceptable and in accordance with literature. Further studies are needed to confirm these results, and to assess the specific role of CRT in this setting.

## SUPPLEMENTARY MATERIALS AND FIGURES



## Abbreviations

ICT: induction chemotherapy
CI: Confidence interval
CRT: Chemoradiotherapy
HR: Hazard ratio
IQR: Interquartile range
NA: Not available
OS: Overall survival
PDAC: Pancreatic duct adenocarcinoma
PFS: Progression-free survival
PS: Performance status
SD: Standard deviation
