# Supplementary material for: Intensification of induction chemotherapy before consolidation chemoradiotherapy improves progression-free survival and time without treatment in patients with locally advanced pancreatic cancers
Source: Oncotarget. 2018 Aug 10;9(62):31999–2009. doi: 10.18632/oncotarget.25877 (PMC6112837; doi:10.18632/oncotarget.25877)
Supplement: Supplementary file 1 [file oncotarget-09-31999-s001.pdf]

Intensification of induction chemotherapy before consolidation chemoradiotherapy improves progression-free survival and time without treatment in patients with locally advanced pancreatic cancers

SUPPLEMENTARY MATERIALS

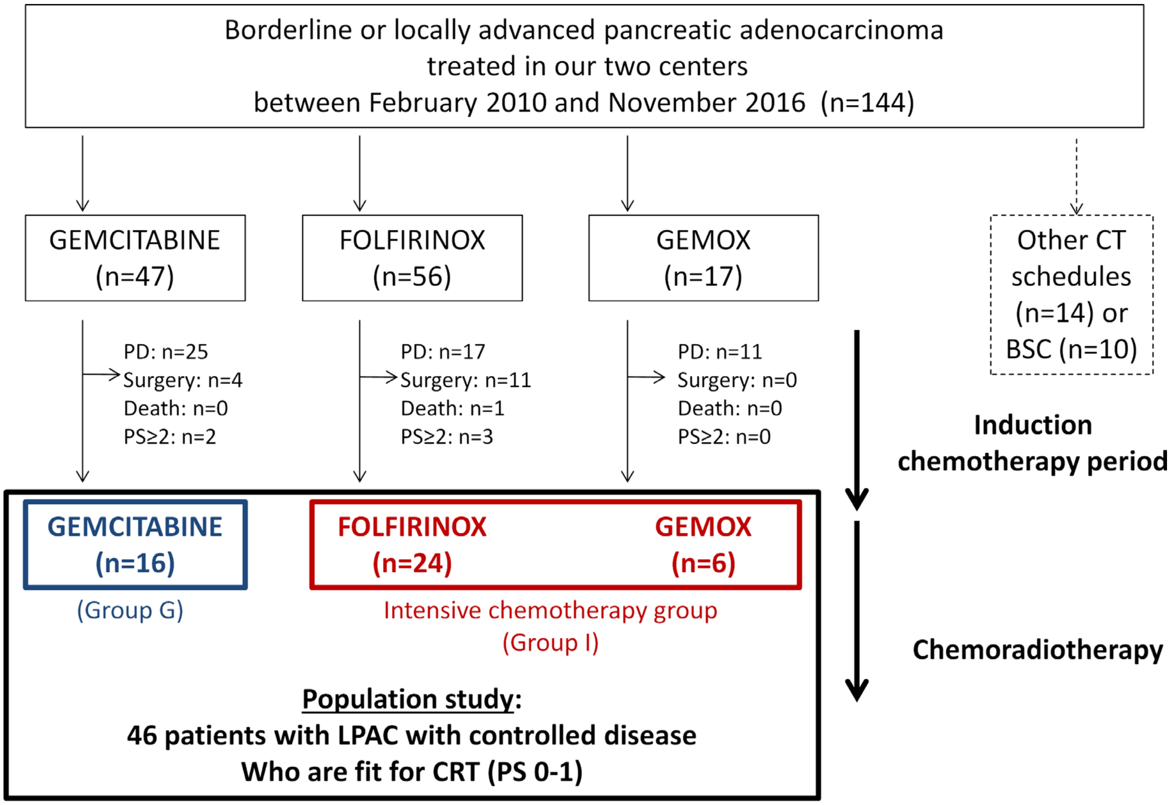

BSC: Best supportive of care; CRT: chemoradiotherapy; CT: chemotherapy; LPAC: locally advanced pancreatic cancer; PD: progression disease; PS: performance status

Supplementary Figure 1: Flowchart of the population study.

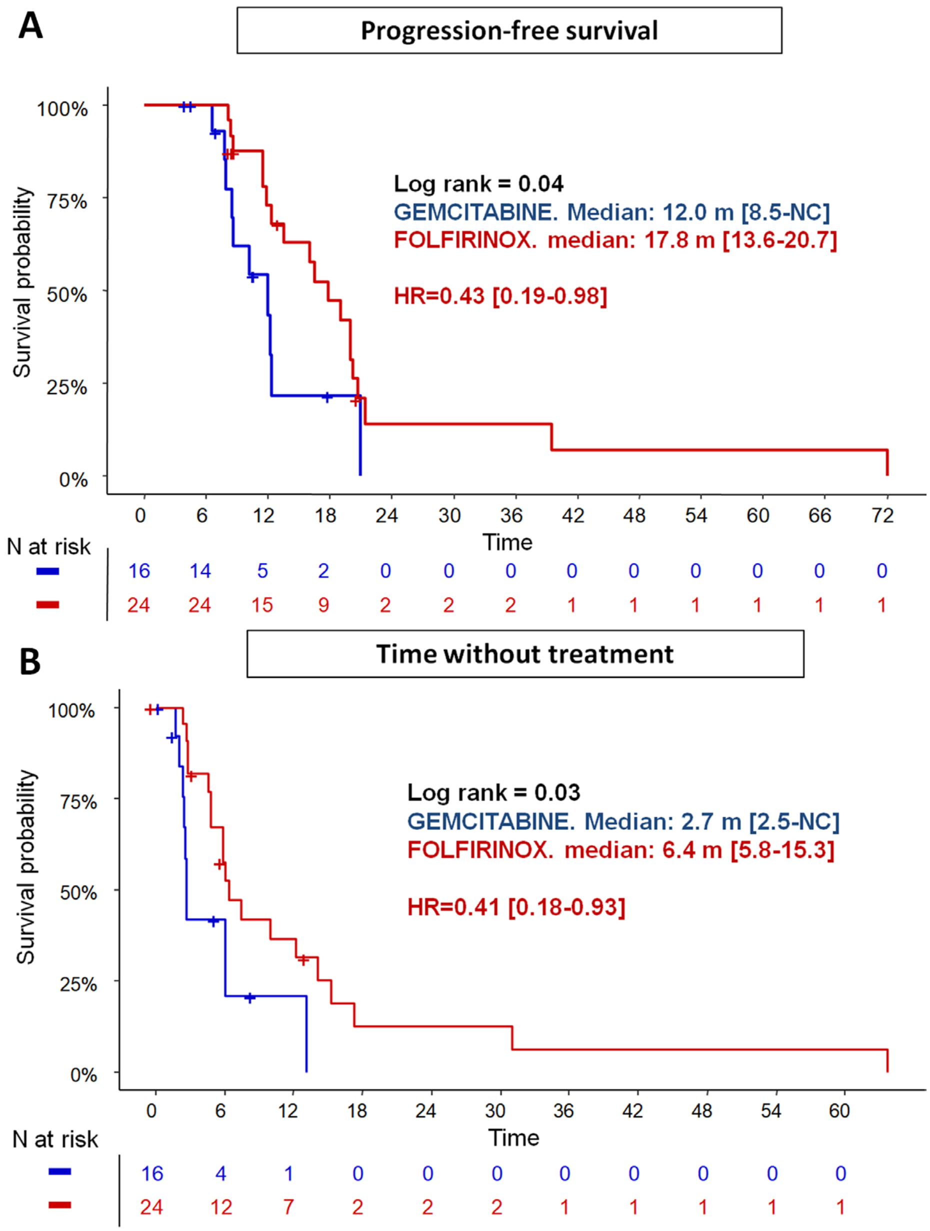

Supplementary Figure 2: Progression free-survival (**A**) and time without treatment (**B**) in patients treated with FOLFIRINOX vs GEMCITABINE

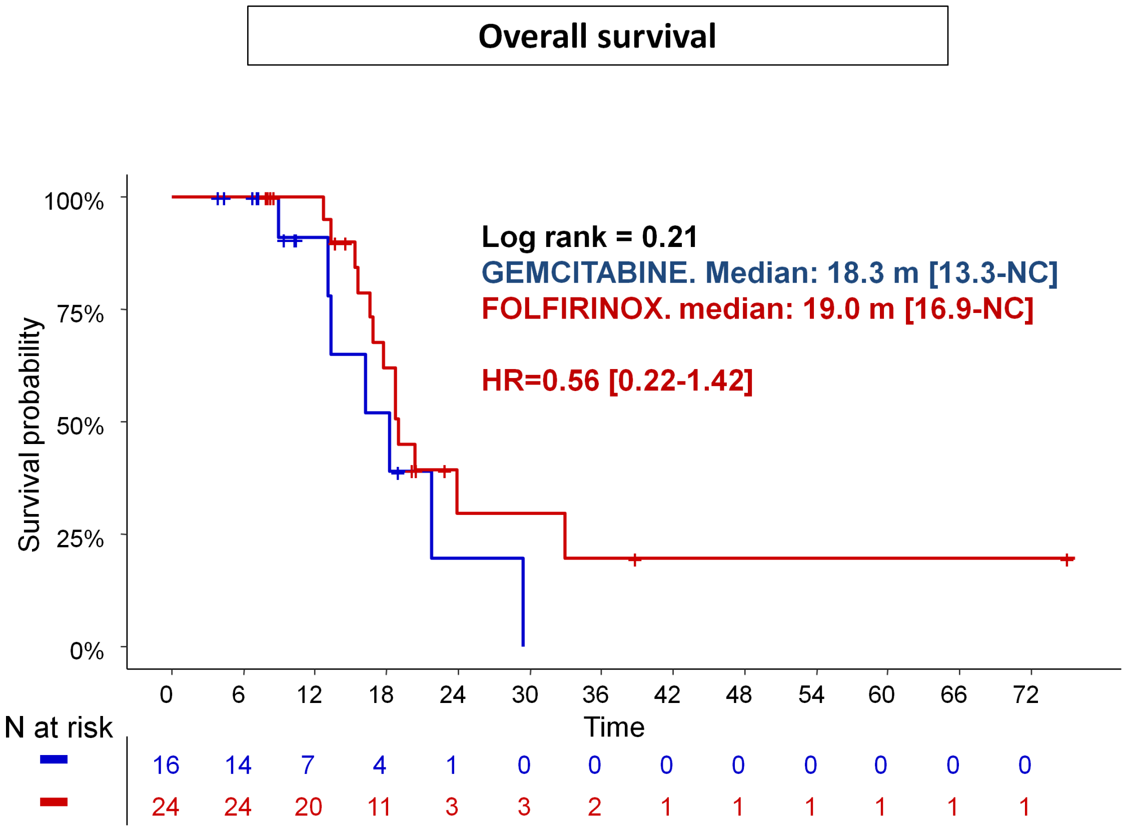

Supplementary Figure 3: Overall survival in patients treated with FOLFIRINOX vs GEMCITABINE.
